# External training load and performance recovery after small-sided games in soccer: Insights for return-to-play management

**DOI:** 10.3934/publichealth.2024016

**Published:** 2024-03-14

**Authors:** Roberto Modena, Federico Schena

**Affiliations:** 1 CeRiSM, Sport Mountain and Health Research Center, University of Verona, Rovereto, Italy; 2 Faculty of Health Sciences and Social Care, Molde University College, Molde, Norway; 3 Department of Neuroscience, Biomedicine and Movement Sciences, University of Verona, Verona, Italy

**Keywords:** football, fatigue, high-intensity, drills, return-to-sport, return-to-performance, injury risk management, acceleration, high-speed running

## Abstract

The return-to-play process' characteristics can vary by injury and sport type but are typically composed of phases of different durations, training targets, and intensities that gradually increase the physiological and mechanical load. In team sports, contact drills are a necessary part of the last phases of this process, and they should be planned using the optimal mechanical load. The present study investigated the external load and kinetic recovery in U19 soccer players performing 6vs6 and 3vs3 small-sided games. A global positioning system (GPS) measured external load metrics. The rate of perceived exertion (RPE) was registered at the end. Total quality of recovery (TQR) was collected at the beginning of the training session and after 24 h. Moreover, before and after the small-sided games (SSGs) and at 24 h, delayed-onset muscle soreness (DOMS) of the legs, sprinting time, and vertical jump height (CMJ) were collected. 6vs6 presented higher values in total distance low-, moderate-, high, and very-high-speed distance, and maximum speed (p < 0.05). However, 3vs3 showed higher number of sprints, acceleration, and deceleration at different intensities. Furthermore, no difference was shown in RPE. The effect of fatigue on sprint seems greater for 6vs6, showing an impairment persistent at 24 h (p < 0.05). Moreover, CMJ height was impaired after 6vs6 and at 24 h (p < 0.05) but did not change after 3vs3 (p > 0.05). DOMS values after SSGs and at 24 h were higher than baseline for both conditions (p < 0.05), while TQR decreased at 24 h in both conditions (p < 0.05). Based on our results, it seems that 6vs6, leading to a greater high-speed running distance, might cause a training load that needs more time to recover. This point may be crucial in a return-to-play process, especially when hamstring muscles are involved.

## 1. Introduction

Soccer clubs face injuries regularly during the season for their senior and youth players [1],[2] with a relevant impact on both technical and economic sides. Studies showed a strong correlation between player match availability and success outcomes such as ranking position, points per match, number of games won, goals scored, and goals conceded [3],[4]. For these reasons, an increasing amount of literature on prevention and return-to-play strategies and procedures has been published over the last decade [5],[6].

After an injury, a return-to-play process starts to bring the player to compete at the pre-injury level as soon as possible. The return-to-play process' characteristics can vary by injury and sport type but are typically composed of phases of different durations, training targets, and intensities that gradually increase the physiological and mechanical load. There is no specific parameter that guides the step from one phase to the next. Still, it is usually regulated by time or specific criteria that ensure the ability and readiness of the players to increase the training load and the playing request [5].

Usually, in a team sport context such as soccer, performing drills with contact is a relevant part of the last phases of the return-to-play process. In this context, one of the more complicated aims of return-to-play practice is the training load prescription and monitoring at the neuromuscular level. When the injured player comes back to the field with the rest of the team, he can be involved in technical-tactical situations and games (e.g., small-sided games) that are characterized by a high rate of complexity, which makes it hard to plan the correct training load (in terms of intensity and volume) for the player returning from the injury.

There is much literature on the physiological and physical responses that small-sided games (SSGs) elicit in soccer players. Several conditions and rules, such as the number of players, the area per player [7]–[9], the presence of the goalkeeper [10],[11], and the use of the coach's feedback [12], have been shown to influence both internal and external training load. On the other hand, a few studies focused on the effect of a small-sided game on neuromuscular fatigue, which might be particularly relevant in modulating the muscular load during the last phases of a return-to-play process. Katis and Kellis [13] compared the effect of a 3vs3 and a 6vs6 game involving young soccer players. The authors showed a decrease in sprint, agility, horizontal jump, and throw-in performance at the end of both small-sided game formats compared to the baseline condition. Moreover, Rebelo et al. [14] showed a decrement in jump performance after both 4vs4 and 8vs8 games compared with baseline conditions tested one week before in college soccer players. On the other hand, the sprint performance seemed to be influenced only after the 4vs4 format. Recently, one study [15] investigated neuromuscular fatigue and kinetics recovery in ten male soccer players after playing two formats of small-sided games. Both small-sided games seemed to affect neuromuscular performance and physiological markers. However, few differences were found between the 4vs4 and 8vs8 formats, where creatine kinase was higher after 48 and 72 hours in the former.

Since spare studies investigated the effect of small-sided game formats on neuromuscular fatigue and recovery kinetics in soccer and the high relevance of these concepts in optimizing training load management and recovery, the present study aimed to investigate the effect of two small-sided game formats on external load, loss of performance and recovery in soccer players. We hypothesized the following: (1) a high number of accelerations and less distance at high speed running in small-sided games played by three players per team (3vs3) compared to those played by six players per team (6vs6), (2) a similar impact of both formats on jump and sprint performance after small-sided games, and (3) quicker neuromuscular recovery after 3vs3 than 6vs6.

## 2. Materials and methods

### 2.1. Procedure

The study was conducted as a cross-sectional design during the 2022/23 season. Data was collected in two similar sessions (3vs3 and 6vs6), each composed of two consecutive days (Day 1+Day 2 and Day 8+Day 9), with a week between and 48 hours after the last match. A small-sided game (3vs3 or 6vs6 in random order) was carried out during Day 1 and Day 8, wearing a GPS device to monitor the external load of players. Total quality of recovery (TQR) was collected at the beginning of Day 1 and Day 8 (Pre) and Day 2 and Day 9 (24h). Moreover, delayed onset muscular soreness (DOMS) value and sprint and vertical jump performance were evaluated at the beginning of Day 1 and Day 8 (pre), after the SSG (post) and at the beginning of Day 2 and Day 9 (24h). Furthermore, at the end of the SSG, the rate of perceived exertion (RPE) was collected. At the beginning of each session, players performed a standardized warm-up (Part 1 and 3 of FIFA 11+).[16] During the week between Day 1 and Day 8, the players trained regularly, and TQR and DOMS collected before Day 1 and Day 8 were compared to check if players were in similar conditions.

### 2.2. Subjects

A power analysis was performed using G*Power (version 3.1.9.4, Düsseldorf University, Düsseldorf, Germany). An expected effect size f = 0.4, α = 0.05, and β = 0.8 indicated a sample size of 12 participants for a repeated-measures design. Twelve male soccer players belonging to a U19 (age 18.3 ± 0.7 years, body mass 73.8 ± 6.1 kg, height 178.0 ± 0.1 cm) professional team took part in the study. Players were typically involved in four training sessions and an official match per week and were used to perform small-sided games during their training sessions. All the participants provided written consent after receiving a complete description of the study protocol and their rights to anonymity. All the procedures were conducted in accordance with the Declaration of Helsinki. Moreover, all the participants were used to perform all the evaluations and the training drills during the usual training sessions with the team.

### 2.3. Small-sided games

A 3-a-side (3vs3) with 100 m^2^ per player (30 m x 20 m) and 6-a-side (6vs6) with 200 m^2^ (60 m x 40 m) were carried out as four bouts of four minutes with two minutes of passive recovery in between on an artificial 3G rubber crumb turf during the first (Day 1) and third session (Day 8). Since there was no goalkeeper, players had to score in a small goal (2 x 1 m) without the offside rule. To maintain high both intensity and motivation, the team compositions were based on the playing position of the players and skill assessment provided by the coach. Moreover, the ball was always available thanks to the presence of several coach collaborators who quickly returned it on the pitch when necessary, and verbal encouragement was provided by the head coach using standardized sentences. The choice of the small-sided game formats and rules has been made based on previous literature, which shows these parameters affect the physical and physiological response of players [11],[12].

### 2.4. Small-sided games external load

During the small-sided games, the players' external load was monitored by a 10-Hz-GPS device (Polar Pro Team, Polar, Finland). The GPS device was connected to a thoracic belt worn by the players 15 minutes before starting the session to maximize the satellite signal's connection. Unfortunately, as the software does not report horizontal dilution of precision (HDOP) and the number of connected satellites, it is not possible to provide these details. Among all the metrics provided by the GPS software, the following were chosen as representative of the training load that may impact the neuromuscular system: total distance covered (TD), distance covered at different speed zones (low-speed distance [0–11 km·h^−1^, LSD], moderate-speed distance [11–15 km·h^−1^, MSD], high-speed distance [15–19 km·h^−1^, HSD] and very high-speed distance [≥19 km·h^−1^, VHSD]), count of accelerations and decelerations performed at different intensities (low-intensity [1–2 m·s^−2^], moderate-intensity [2–3 m·s^−2^] and high-intensity [ >3 m·s^−2^]), number of sprints and maximal speed. A previous study has demonstrated a moderate to good intra-unit reliability of this system in measuring distance at different speeds [17].

### 2.5. Perception scales

TQR rate was collected at the beginning of each session to evaluate the recovery state of the players [18]. Values on Day 1 and Day 3 were compared to ensure that players presented a similar state between the conditions. On the other hand, rates on Day 2 and Day 4 were compared to assess the effect of the SSG format on recovery at 24 hours.

Delayed onset muscular soreness at the lower limb was evaluated using a 100 mm visual analogue scale (VAS-DOMS) [19]. Moreover, RPE was collected at the end of the SSG using the CR-100 Borg Scale [20] to quantify the internal load of the players. All the scales were presented individually to each player following the suggested procedure to increase the validity and reliability of the measure.

### 2.6. Performance tests

Players performed three trials sprinting 30 meters, while time on 10 (10m-sprint), 20 (20m-sprint) and 30 (30m-sprint) meters were taken by a photocells system (Polifemo, Microgate, Bolzano, Italy) which presents high reliability (CV < 2%) [21]. Vertical jump performance was evaluated by measuring the flying time during a countermovement jump using the Optojump system (Microgate, Bolzano, Italy); during the jump, the players kept their hands at the hip. A passive recovery of 30 seconds was observed between the three trials. This test was proven to have excellent reliability (CV = 2.2%) [22].

### 2.7. Statistical analysis

The normal distribution of data was checked using the Kolmogorov-Smirnov test. Total distance, the distance at different speeds, and RPE were compared between the two SSGs using a paired T-test. As distribution was not normal, maximal speed and the number of accelerations, decelerations, and sprints were compared by means of the related-samples Wilcoxon signed rank test. Profile of performance in 30-m sprint and CMJ, DOMS, and TQR were analyzed using a two-way repeated measure ANOVA with time and condition as independent factors. A pairwise comparison with least significance difference adjustment was performed when an effect (p < 0.05) was found. Hedges' g was used as standardized effect size [23] and interpreted qualitatively using Cohen's benchmarks [24].

## 3. Results

There is no evidence of difference (p > 0.05) between the two conditions at pre for TQR and DOMS, indicating similar recovery state of players. Data comparisons of external load parameters and RPE are shown in Table 1. The 6vs6 format presented higher values in TD, moderate-, high- and very high-speed running distance and maximum speed, whereas 3vs3 showed a higher number of sprints, acceleration and deceleration at different intensities and more distance spent at low speed. However, no difference was shown in RPE.

**Table 1.** External load metrics and RPE measured in 3vs3 and 6vs6 small-sided games.

	**3vs3**	**6vs6**	**Mean difference (95% CI)**	**P value**	**Effect size Hedge's g**
	**Mean (SD)**				
*Total distance [m]*	1955.4 (219.6)	2230.9 (226.4)	−274.6 (−344.1 to −205.0)	<0.001	1.14 (0.24 to 2.04) Large
*Low-speed running [m]*	1150.7 (91.2)	1040.1 (121.9)	110.6 (49.1 to 172.2)	0.003	−0.95 (−1.83 to −0.07) large
*Moderate-speed running [m]*	375.4 (107.6)	525.2 (142.6)	−149.7 (−224.5 to −75.0)	0.037	1.09 (0.20 to 1.99) Large
*High-speed running [m]*	222.9 (107.8)	315.6 (114.2)	−92.6 (−141.8 to −43.5)	0.004	0.77 (−0.10 to 1.64) Moderate
*Very high-speed running [m]*	169.7 (133.3)	321.9 (192.4)	−152.2 (−228.9 to −75.4)	0.002	0.85 (−0.02 to 1.72) Large
*Maximum speed [km(h^−1^]*	26.2 (2.0)	27.8 (1.8)	−1.66 (−3.0 to −0.37)	0.026	0.78 (−0.09 to 1.74) Moderate
*Sprints [n]*	29.8 (12.3)	23.0 (10.1)	6.8 (2.2 to 11.5)	0.016	−0.56 (−1.41 to 0.29) Moderate
*Low-intensity acceleration [n]*	101.1 (12.3)	80.9 (10.0)	20.2 (10.9 to 29.4)	0.004	−1.66 (−2.63 to −0.69) Large
*Moderate-intensity acceleration [n]*	38.9 (9.9)	29.3 (5.5)	9.6 (5.2 to 14.0)	0.007	−1.11 (−2.00 to −0.21) Moderate
*High-intensity acceleration [n]*	24.0 (10.6)	19.5 (8.9)	4.6 (0.8 to 8.3)	0.028	−0.42 (−1.27 to 0.42) Small
*Low-intensity deceleration [n]*	97.1 (9.5)	74.8 (5.8)	22.3 (13.7 to 30.8)	0.004	−2.62 (−3.75 to −1.48) Large
*Moderate-intensity deceleration [n]*	40.0 (8.3)	32.5 (6.8)	7.6 (2.9 to 12.2)	0.011	−0.91 (−1.79 to −0.03) Large
*High-intensity deceleration [n]*	20.7 (13.0)	14.3 (8.0)	6.5 (1.2 to 11.7)	0.016	−0.55 (−1.40 to 0.30) Moderate
*RPE*	5.5 (1.5)	5.3 (0.7)	0.2 (−0.8 to 1.3)	0.328	−0.16 (−0.99 to 0.68) Trivial

Note: The sprint and CMJ performance at pre, post, and 24h are presented in Table 2 and Figure 1. Both 3vs3 and 6vs6 seemed to negatively impact the sprint performance immediately after the small-sided game for all the measures; the performance impairment resulted in recovery at 24h in both conditions (no significant differences with Pre, p > 0.05), but it seems better in 3vs3 (significant difference with Post, p < 0.05). Moreover, CMJ height was impaired after 6vs6 and at 24h (p < 0.05) but did not change after 3vs3 (p > 0.05) and improved at 24h in this condition (p < 0.05).

**Table 2.** Performance and perceptual data measured before, after and 24h the small-sided games.

		**Mean (SD)**			**P value /Effect size Hedge's g (95% CI)**		
		**Pre**	**Post**	**24h**	**Pre vs Post**	**Post vs 24h**	**Pre vs 24h**
**10m-sprint [s]**	**3vs3**	1.83 (0.10)	1.85 (0.09)	1.81 (0.08)	0.047 0.20 (−0.61 to 1.00)	0.020 −0.44 (−1.25 to 0.37)	0.255 −0.21 (−1.01 to 0.60)
	**6vs6**	1.79 (0.06)	1.83 (0.07)	1.79 (0.09)	0.773 0.57 (−0.25 to 1.39)	0.088 −0.46 (−1.27 to 0.35)	0.773 <0.00 (−0.80 to 0.80)
**20m-sprint [s]**	**3vs3**	3.08 (0.13)	3.13 (0.11)	3.04 (0.1)	0.005 0.39 (−0.42 to 1.19)	0.033 −0.80 (−1.63 to 0.03)	0.202 −0.32 (−1.13 to 0.48)
	**6vs6**	3.04 (0.09)	3.09 (0.1)	3.02 (0.17)	0.001 0.49 (−0.32 to 1.30)	0.058 −0.47 (−1.28 to 0.34)	0.514 −0.14 (−0.94 to 0.66)
**30m-sprint [s]**	**3vs3**	4.25 (0.16)	4.32 (0.15)	4.21 (0.13)	<0.001 0.42 (−0.39 to 1.23)	<0.001 −0.73 (−1.56 to 0.10)	0.055 −0.26 (−1.06 to 0.55)
	**6vs6**	4.21 (0.12)	4.28 (0.13)	4.24 (0.14)	<0.001 0.52 (−0.29 to 1.33)	<0.001 −0.28 (−1.08 to 0.53)	0.075 0.21 (−0.59 to 1.02)
**CMJ [cm]**	**3vs3**	35.89 (5.93)	37.03 (4.86)	38.39 (5.81)	0.103 0.20 (−0.61 to 1.00)	0.053 0.24 (−0.57 to 1.04)	0.001 0.40 (−0.41 to 1.20)
	**6vs6**	37.87 (5.2)	36.32 (5.24)	36.14 (5.77)	0.038 −0.28 (−1.08 to 0.53)	0.793 −0.03 (−0.83 to 0.77)	0.021 −0.29 (−1.10 to 0.51)
**DOMS [mm]**	**3vs3**	17.73 (17.54)	33.18 (21.31)	25.36 (18.21)	0.002 0.74 (−0.09 to 1.56)	0.028 −0.37 (−1.17 to 0.44)	0.040 0.40 (−0.41 to 1.21)
	**6vs6**	14.82 (10.79)	33.55 (15.69)	24.09 (16.57)	<0.001 1.29 (0.41 to 2.17)	0.009 −0.55 (−1.36 to 0.27)	0.015 0.62 (−0.20 to 1.44)
**TQR**	**3vs3**	16.09 (2.84)		14.09 (2.02)			0.013 −0.75 (−1.58 to 0.07)
	**6vs6**	16.18 (2.18)		13.64 (1.69)			0.002 −1.21 (−2.08 to −0.34)

**Figure 1.**
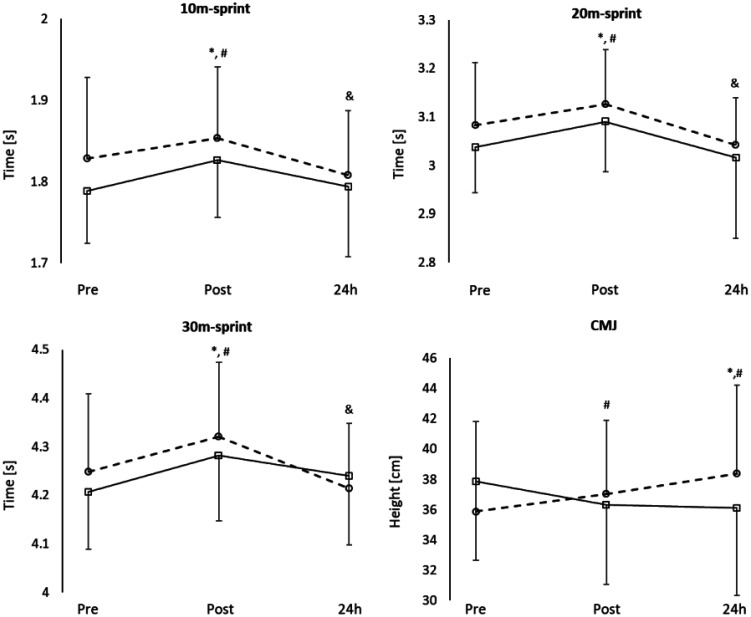
Recovery kinetics of sprint and CMJ performance. Circle and dashed line represent 3vs3, Square and Continue line represent 6vs6. *difference with pre in 3vs3, p < 0.05; ^#^difference with pre in 6vs6, p < 0.05; ^&^difference with post in 3vs3, p < 0.05.

**Figure 2.**
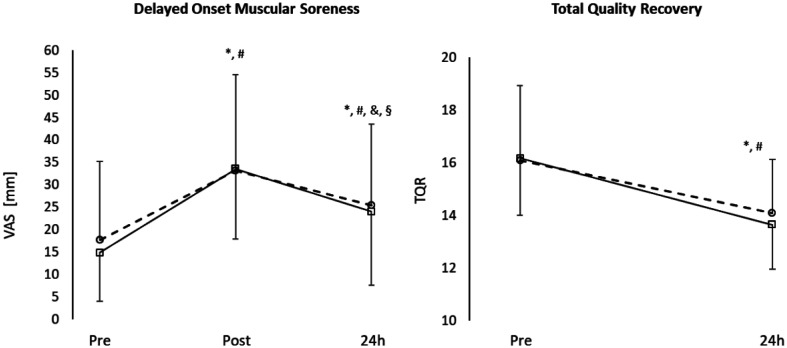
Recovery kinetics of perceptual parameters. Circle and dashed line represent 3vs3, square and continue line represent 6vs6. *difference with pre in 3vs3, p < 0.05; #difference with pre in 6vs6, p < 0.05; &difference with post in 3vs3, p < 0.05; ^§^difference with post in 6vs6, p < 0.05.

Perception of fatigue and recovery data are shown in Table 2 and Figure 2. DOMS values after SSGs and at 24h were higher than baseline for both conditions (p < 0.05), while TQR decreased at 24h in both conditions (p < 0.05).

## 4. Discussion

This study aimed to compare the external load and the effect on performance recovery of two formats of small-sided games (6vs6 and 3vs3) played by twelve U19 male soccer players during their in-season training sessions.

### 4.1. External load during SSGs

Our results showed higher total (+14%), moderate- (+40%), high- (41.8%), and very-high speed (89.4%) running distances covered and maximal speed (+9.5%) reached in 6vs6 compared to 3vs3. However, during 3vs3, players covered higher low-speed running distance (+10.6%), performed a higher number of accelerations (low- +25%, moderate +32.8%, and high intensity +23.6%), decelerations (low- +29.8%, moderate +23.4%, and high intensity +45.5%), and sprints (+29.6%) than during 6vs6. Despite of these differences, RPE was similar for the two conditions. Some of these data agree substantially with the literature that showed higher total distance and distance covered at high-speed running when the area per player increases [9],[11],[12]. On the other hand, data on accelerations and sprints are less consistent; we found that a greater number of accelerations, decelerations, and sprints happen with a higher density of players (100 m^2^ vs 200 m^2^ per player). In contrast, Hodgson et al. [7] did not show any differences in acceleration profile comparing 5vs5 in two similar area-per-player conditions (120 m^2^ vs 200 m^2^). Moreover, Riboli et al. [9] reported no clear relationship between area per player and distance covered accelerating during different drills. However, Rebelo et al. Rebelo et al., 2016) showed a higher number of high-intensity accelerations and decelerations in 4vs4 than in 8vs8 small-sided games. Furthermore, Gaudino et al. [25] observed an increase in the number of accelerations as the pitch's dimension and players' number decreased.

### 4.2. Neuro-muscular load

Combining these data, it seems to appear a double scenario regarding neuromuscular load in small-sided games. A lower number of players and small pitch's size seem to lead to a high number of accelerations, while when the pitch's area increases, there is a greater distance covered at high- and very-high speed running. Both of these neuromuscular loads (i.e., accelerations and high-speed running distance) are useful in a regular training season and a return-to-play process Still, they may have different kinetic recovery and this must be studied.

### 4.3. Neuromuscular fatigue and recovery kinetics

Coaches commonly use these situational drills to train both physical and technical-tactical characteristics. Moreover, they are a necessary part of a return-to-play process when a player approaches to return to train and play with the rest of the team. In this context, coaches must know the load on the skeletal muscle system and understand the mechanisms behind muscular fatigue recovery after small-sided games to avoid overload and re-injury episodes. The importance of monitoring external load in this context was highlighted in a recent meta-analysis [26] that pointed out the presence of moderate to strong relationship between distance covered above 19.8 km/h and acute and residual fatigue after a soccer match. Furthermore, Teixeira et al. have shown the presence of a relationship between external training load and recovery status in youth soccer players [27]. Moreover, the same research group reported that training load and recovery status in this population may be influenced by age and other factors [28].

Our results showed that the impairment of sprint performance immediately after the exercise and the recovery at 24h are similar in both conditions for 10-m and 20-m sprints, but when we consider the longer distance (30-m sprint) it seems that 6vs6 induces a worse recovery than 3vs3; this difference might be explained by the higher amount of high- and very-high speed running distance covered in the 6vs6 drill which may reasonably impact on sprint performance in longer rather than in the shorter distances. This may be explained by a condition of fatigue of hamstring more pronounced after small-sided games with larger area-per-player [29]. Similarly, the countermovement jump performance seemed impaired only after the 6vs6 and it was still impaired at 24h in this condition but not in the 3vs3. These results partially agree with Papanikolaou et al. [15] who compared 4vs4 and 8vs8 small-sided games in adult soccer players and found slight differences in sprint and CMJ recovery profiles. The 30-m sprint time increased at 24h and 48h in both conditions but only in 8vs8 at 72h. The CMJ performance decreased after the exercise, at 24h and at 48h in 8vs8, while it was impaired only at 24h in 4vs4. Moreover, in the study of Rebelo et al. [14], the authors showed that the sprint performance on 5m and 15m distances was impaired after the 4vs4 and not after the 8vs8 condition. However, Katis and Kellis [13], observed a similar effect of 3vs3 and 6vs6 in impairing sprint, agility and jump performance in young soccer players immediately after exercise. All of these results are not always consistent, and it can depend on the age of participants and the structure of the sessions; the participants in the study of Katis and Kellis were 13 years old and comparing their neuromuscular responses with those of adult participants can be biased by biological differences. Furthermore, in the Rebelo et al. study, the 4vs4 small-sided games were organized in two sets of 3 x 6 minutes of play with 3 minutes of recovery between repetitions and 5 minutes between sets, whereas the 8vs8 was performed as two halves of 18 minutes. This different organization of exercise and recovery time may influence the results.

In our study, the behavior of the perception of recovery seemed to be similar between 4vs4 and 8vs8. Total quality recovery rate decreased at 24h compared to pre, while VAS DOMS increased immediately after exercise and tended to improve at 24h without reaching the pre values. In the literature, only Papanikolaou et al. assessed some parameters of muscle damage perception, showing more persistent muscle fatigue in 4vs4 compared to 8vs8. Interestingly, this data was not consistent with the kinetics recovery of sprint and CMJ performance, confirming the absence of a relationship between subjective and objective measures in athlete monitoring [30].

## 5. Limitation

The area-per-player and number of players are two factors that may influence the physical, physiological, and perceptual response in different ways. In this study, these factors were not considered separately, which may represent a limitation. Moreover, although the responses and recovery kinetics described in our study may be useful in a return-to-play process, they are assessed in not-injured players and it should be considered.

## 6. Conclusions

Based on these findings, it seems larger area-per-player and a medium number of players (e.g., 6vs6), leading to a larger amount of high-speed running distance, might cause a training load that needs more time to recover. Weekly periodization should consider these differences because one of the coach's aims is to promote an optimal recovery for increasing the performance and reducing the risk of injuries during the match day. Thus, small-sided games with high player density might be proposed closer to the match day than those with low player density. Otherwise, when the goal is to support a player during the last phases of the return-to-play process, adequate recovery is a key factor to increase the performance of the player limiting the risk of overload and injury recurrence. Considering the relevant impact of small-sided games played with low player density on hamstring fatigue and recovery, this point may be crucial, especially when hamstring muscles are injured.

## Use of AI tools declaration

The authors declare they have not used Artificial Intelligence (AI) tools in the creation of this article.
